# Establishment of Primary Cell Cultures from Canine Oral Melanomas via Fine-Needle Aspiration: A Novel Tool for Tumorigenesis and Cancer Progression Studies

**DOI:** 10.3390/ani14131948

**Published:** 2024-07-01

**Authors:** Adriana Lo Giudice, Ilaria Porcellato, Martina Pellegrini, Sven Rottenberg, Chang He, Alfredo Dentini, Giulia Moretti, Monica Cagiola, Luca Mechelli, Elisabetta Chiaradia, Chiara Brachelente

**Affiliations:** 1 Department of Veterinary Medicine, University of Perugia, Via San Costanzo 4, 06126 Perugia, Italy; adriana.logiudice@live.it (A.L.G.); giulia.moretti@unipg.it (G.M.); luca.mechelli@unipg.it (L.M.); elisabetta.chiaradia@unipg.it (E.C.); chiara.brachelente@unipg.it (C.B.); 2 Istituto Zooprofilattico Sperimentale dell’Umbria e delle Marche “Togo Rosati”, Via G. Salvemini 1, 06126 Perugia, Italy; m.pellegrini@izsum.it (M.P.); m.cagiola@izsum.it (M.C.); 3 Institute of Animal Pathology, Vetsuisse Faculty, University of Bern, Länggassstrasse 120, 3012 Bern, Switzerland; sven.rottenberg@unibe.ch (S.R.); chang.he@unibe.ch (C.H.); 4 Clinica Veterinaria Tyrus, Strada delle Campore 30L, 05100 Terni, Italy; alfredo.dentini@gmail.com

**Keywords:** melanocytes, oral melanoma, fine-needle aspiration, primary cell culture, dog

## Abstract

**Simple Summary:**

Oral melanomas are the most common oral tumors in dogs. They are usually aggressive, invasive, and bear a poor prognosis. These neoplasms have genetic and biological similarities with human oral melanoma, suggesting the potential use of the dog as a model in comparative studies. Primary two- and three-dimensional cell cultures from spontaneously arising canine oral melanocytic tumors and their nodal metastasis are established in this study, starting from cells sampled by fine-needle aspiration (FNA). This technique could be considered a helpful and less invasive method to collect samples for cell cultures, particularly for cases where surgery cannot be performed or when the owner refuses a surgical approach. This cell culture model contributes to the array of in vitro models, providing valuable tools for characterizing neoplastic cells and investigating the cellular pathways supporting cancer progression and invasion.

**Abstract:**

Oral melanomas are the most common oral malignancies in dogs and are characterized by an aggressive nature, invasiveness, and poor prognosis. With biological and genetic similarities to human oral melanomas, they serve as a valuable spontaneous comparative model. Primary cell cultures are widely used in human medicine and, more recently, in veterinary medicine to study tumorigenesis, cancer progression, and innovative therapeutic approaches. This study aims to establish two- and three-dimensional primary cell lines from oral canine melanomas using fine-needle aspiration as a minimally invasive sampling method. For this study, samples were collected from six dogs, represented by four primary oral melanomas and five lymph nodal metastases. The cells were digested to obtain single-cell suspensions, seeded in flasks, or processed with Matrigel^®^ to form organoids. The cell cultures were characterized through flow cytometry using antibodies against Melan-A, PNL2, and Sox-10. This technique offers a minimally invasive means to obtain cell samples, particularly beneficial for patients that are ineligible for surgical procedures, and enables the establishment of in vitro models crucial for comparative studies in mucosal melanoma oncology. To the best of our knowledge, this is the first work establishing neoplastic primary cell cultures via fine-needle aspiration in dogs.

## 1. Introduction

Melanocytic tumors are the most common malignant neoplasms of the oral cavity in dogs [1,2,3]. In particular, canine oral mucosal melanomas (OMMs) typically display aggressive behavior, invade surrounding tissues readily, and have a propensity for metastasis to regional lymph nodes as well as distant sites [3,4,5,6]. These neoplasms are often detected at an advanced stage with considerable tumor extension. Their invasive growth often leads to profound penetration—frequently associated with bone lysis—rendering the primary neoplasm frequently inoperable, especially in the presence of distant metastases [5], thereby contributing significantly to the poor prognosis associated with oral melanoma [5,6]. Nowadays, different therapeutic approaches are available to treat this tumor, with surgery remaining the cornerstone [7]; however, in cases where extensive tumor growth hinders surgical intervention, alternative local treatments such as radiotherapy and electrochemotherapy have demonstrated efficacy in reducing tumor size [7,8]. Immunotherapy, which bolsters the immune response against the neoplasm or targets specific molecular pathways associated with tumor progression and metastasis, is showing promising results in the treatment of different tumors in both humans and dogs [9,10]. This therapeutic approach can be pursued through vaccination, electrovaccination, gene therapy, and checkpoint inhibitors [7,11,12,13]. Nevertheless, the limited availability of commercially available drugs for dogs and the predominant reliance on in vitro models in research present challenges [14].

While in vitro models are extensively employed in human medicine, studies on canine OMM, especially those centered on three-dimensional cell cultures, are limited [15]. This is attributed to the recent realization of dogs’ significance as spontaneous models for studying human melanomas, especially oral ones, which share several traits with their human counterparts including responses to similar immunotherapeutic approaches [14,16,17,18,19,20]. In addition, research on the immune environment of canine OMM has expanded, offering valuable insights into these tumors [21,22,23,24,25]. The rarity of mucosal melanoma in humans accounts for fewer possibilities of performing clinical studies in large groups of patients but the availability of a spontaneous animal model could represent a significant advantage. Establishing 3D cell cultures, potentially incorporating specific immune cell co-cultures, would provide an important tool to study interaction mechanisms between neoplastic and immune cells and test new drugs against mucosal melanomas.

Unfortunately, establishing cell lines from surgical excision in canine OMM can pose challenges, often due to the poor clinical condition of the affected dogs or the ineligibility of the tumor for surgery. Similarly, obtaining an incisional biopsy is sometimes challenging because owners opt for minimally invasive procedures. Furthermore, when cytology results along with staging confirm metastatic melanoma, owners may not consent to further procedures, including incisional biopsies. Additionally, patients are often referred to oncology centers after relapse or following surgeries performed by other practitioners. In the former scenario, owners frequently decline further treatments, while in the latter, only the metastasis can be sampled if present. Consequently, FNA emerges as a valuable tool for obtaining sufficient cells to initiate primary cell cultures, facilitating the creation of both 2D and 3D models. FNA sampling is a procedure often performed for diagnostic purposes and it is frequently carried out during collateral diagnostic exams, such as X-rays or CT scans, which are necessary to evaluate the tumor origin and stage. Furthermore, all these procedures necessarily require sedation of the dog, thus allowing for safer manipulation.

This technique has found successful application in human medicine for various tumor types, particularly in the establishment of three-dimensional cell cultures [26,27,28,29,30,31]. This approach can also offer a means for personalized disease characterization tailored to each patient’s condition.

This study aims to establish two-dimensional and three-dimensional cell lines of canine OMM, using FNA as a sampling method. More specifically, three-dimensional cell cultures will include spheroids and organoids formation. The former are spherical cultures established starting from a two-dimensional culture without a scaffold, while the latter are directly set up with a scaffold from the digested sample and can be considered a more complex unit. The use of FNA could be considered a helpful technique to implement the collection of viable neoplastic cells. Additionally, these in vitro models can serve as invaluable tools for investigating different oncological aspects, spanning from elucidating molecular pathways associated with tumor progression and metastasis formation to unraveling the complexities of the tumor microenvironment.

## 2. Materials and Methods

### 2.1. Reagents

DMED/Ham’s F12 medium (10-090-CV) and Matrigel^®^ (CLS354230) were obtained from Corning^®^, New York, NY, USA;

Penicillin/Streptomycin (ECB3001D), Amphotericin B (ECM0009D), Gentamycin (ECM0011B), and Fetal Bovine Serum (FBS) (ECS5000L) were purchased from Euroclone, Pero, Italy;

Collagenase I (C0130) and 0.025% trypsin (C-41012) were ordered from Sigma-Aldrich, St. Louis, MO, USA;

Melanoma-associated antigen (PNL2) (sc-5950306), PNL2-Alexa Fluor 488 antibody (sc-59306), and Sox-10 antibody, (sc-365692) were bought from Santa Cruz Biotechnology, Santa Cruz, CA, USA;

Melan-A (a103-m27c10-m29e3), Melan-A Alexa Fluor 594 antibody (a103-m27c10-m29e3), Mouse and Rabbit Specific HRP Detection IHC Kit (ab93677), and 3-amino-9-ethylcarbazole chromogen (AEC Substrate System) (ab64252) were purchased from Abcam, Cambridge, UK;

Ki-67 antibody (clone MIB-1) was obtained from Dako, Denmark;

Tyrosinase-related protein 1—TRP-1 (ls-b4011) from LSBio, Lifespan Biosciences, Lynwood, Washington, DC, USA;

LEUCOPERM reagents (buf09B) and LYNX Rapid RPE-Cy7 Antibody Conjugation Kit (lnk111pecy7) were bought from Bio-Rad, Hercules, CA, USA.

### 2.2. Sample Collection

This study was conducted following the ethical approval of the University of Perugia (Ethical approval number n. 18/2022). Samples for this study were collected from OMMs of sedated patients, after owner consent. Inclusion criteria comprised: (a) prior cytological, histological, or immunohistochemical diagnosis of oral melanoma (using Melan A, PNL2, and Sox-10 antibodies) [6,32,33,34,35,36,37], with or without associated lymph node metastasis; (b) tumor diameter > 1 cm^2^; (c) no previous anti-neoplastic therapies (i.e., chemotherapy or electrochemotherapy). The histologic criteria for the inclusion of the tumor as a melanoma in our study were as follows: moderate to marked cellular atypia, more than 4 mitoses in 2.37 mm^2^, infiltrative growth, and vascular invasion. If the tumor was poorly differentiated, immunohistochemistry was performed to characterize it and the diagnosis of melanoma was confirmed when the neoplastic cells were positive for Melan-A or PNL2, and Sox-10 [6,34,36].

Samples were collected from hospitals with an oncology service; hence, patients were often referred from other facilities where the surgeries and histological diagnostic evaluations were previously conducted.

Samples were collected using a 25-gauge sterile needle attached to a 5 mL sterile syringe. The needle was inserted perpendicularly into the nodule or the metastatic lymph node, applying backward pressure through the syringe holder, followed by rapid release. This sequence of movements was repeated 10–20 times without removing the needle from the mass but shifting it laterally, thus sampling an area within a few millimeters. Depending on the size and the consistency of the tumor, this process was repeated (up to 5 times), with the needle inserted into different areas. Subsequently, the material was immediately expelled into 2 mL sterile tubes containing complete medium consisting of DMED/Ham’s F12 medium supplemented with Penicillin/Streptomycin (100 U/L; 100 µg/mL), Amphotericin B (2.5 µg/mL), Gentamycin (50 µg/mL), and 20% FBS. The collected samples were then processed within 2 h.

### 2.3. Cell Culture

The collected samples were washed three times in medium without FBS and the collagenous stroma was digested with 2 mg/mL collagenase I for 1 h at 37 °C. The enzymatic activity was halted by adding complete medium and cells were collected via centrifugation (at 340× *g* for 7 min).

To establish two-dimensional cell cultures, cells were seeded at the density of 2 × 10^4^ cells/cm^2^ and then cultivated in complete medium at 37 °C in a humidified atmosphere containing 5% CO_2_ until reaching 80/90% confluency. The culture medium was changed every 48 h.

To establish organoids, 8 × 10^4^ cells were suspended in 40 μL of complete medium with 70% Matrigel^®^ and were gently seeded in the wells of the pre-warmed 24-well plate, forming a drop. The 24-well plate was incubated at 37 °C for 5 min followed by a plate inversion for an additional 10 min to optimize the drop shape. After that, the plate was reverted again for 20 min, the complete medium was added, and cells were incubated at 37 °C in a humidified atmosphere containing 5% CO_2_. The culture medium was changed every 48 h. The organoids were expanded when the drop exhibited a high cellular density.

To establish spheroids, cells obtained from 2D cultures were used, following the method described by Saraiva et al. [38]. Briefly, the 96-well plate round bottom was coated with 50 μL of 1.5% sterile agarose. After cooling, 1 × 10^3^ cells resuspended in 200 μL of complete medium were added to each plate well and incubated at 37 °C in a humidified atmosphere containing 5% CO_2_.

### 2.4. Cell Characterization

Flow cytometry was performed to confirm the melanocytic cellular phenotype in cases 1, 2, 3, and 6. Cases 4 and 5 did not reach a sufficient yield to proceed with the analysis.

The technique was carried out on two-dimensional primary cell cultures. Upon reaching 80/90% confluency, cells were collected and then aliquoted into flow cytometry tubes at the density of 1 × 10^6^ cells/100 μL. Subsequently, these cells were treated with LEUCOPERM reagents for permeabilization, according to the manufacturer’s protocol.

Samples were labeled with 10 μL final volume of anti-Melan-A Alexa Fluor 594 antibody, anti-PNL2-Alexa Fluor 488 antibody, and anti-Sox-10 antibody, conjugated in-house with a LYNX Rapid RPE-Cy7 Antibody Conjugation Kit.

Antibodies were diluted at the optimal concentration (according to the manufacturer’s instructions) in dilution buffer immediately before use. Following centrifugation, cells were resuspended in 400 µL of sheet fluid for the flow cytometric acquisition using BD FACS Canto II equipped with two lasers (488 nm and 640 nm) and with BD FACSDivaTM Software (version 4.2). Acquisition parameters were set at FSC 429 V, SSC 341 V, AF488 264 V, AF594 330 V, PE-Cy7 401 V. Data were processed with Kaluza analysis software vers. 2.1.

In the organoid culture of case 2, hematoxylin and eosin staining and immunohistochemistry were performed to evaluate the morphological and phenotypical aspects of these cultures.

Organoids were collected gently by disrupting the drop and centrifuged in a 1.5 mL tube (7 min at 340× *g*). The supernatant was discarded and the pellet was processed following the method described by Yoshimoto et al. [39]. Hence, the pellet was fixed for 20 min at room temperature adding 4% paraformaldehyde (pH 7.2), and then dehydrated using a series of increasing concentrations of alcohol. Subsequently, the pellet was embedded in paraffin and the formalin-fixed and paraffin-embedded (FFPE) organoids obtained were processed routinely.

The antibodies used for the phenotypical characterization on immunocytochemistry were against Melan-A, PNL2, Sox-10, and TRP-1. Ki-67 was used to assess the proliferation index. Four-micron sections were cut from each FFPE sample, mounted on polarized slides, and dried. After dewaxing, antigen retrieval was performed according to the antibody manufacturer’s instructions. Peroxidase and protein block followed and, then, the diluted antibodies were applied to the sections according to the manufacturer’s instructions, followed by a 2-hour incubation period. Following a rinse, a secondary biotinylated goat anti-polyvalent antibody was applied for 10 min. Finally, to reveal the immune complexes, the slides were incubated with peroxidase-labeled streptavidin for 10 min and then with AEC. Carazzi’s hematoxylin was used as a counterstain.

## 3. Results

### 3.1. Case Study

**Figure 1:**
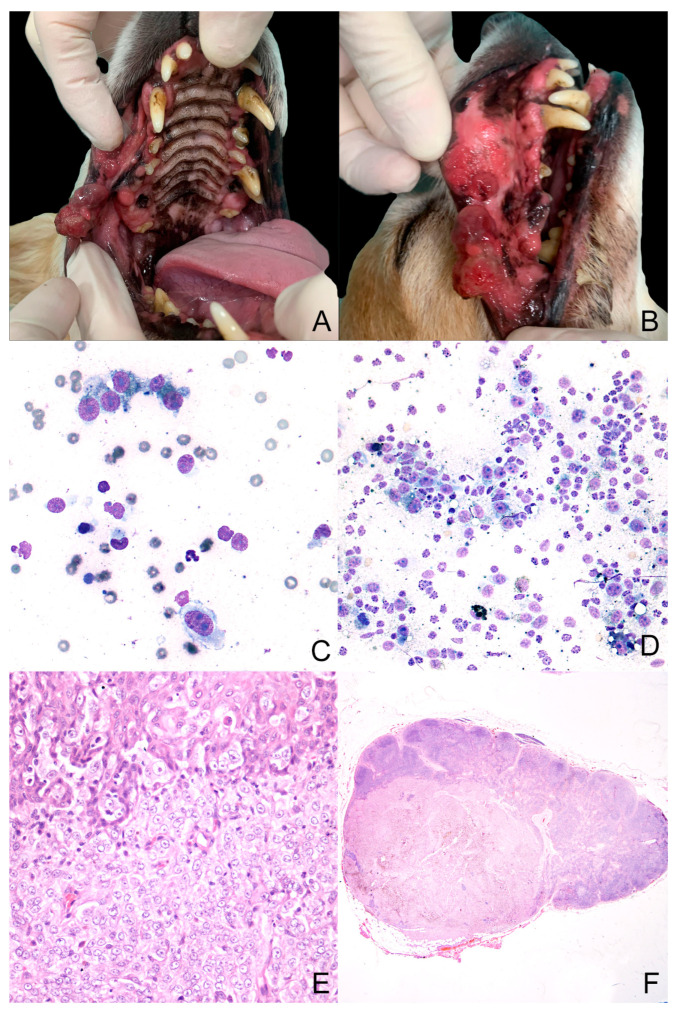
Case 3 (**A**–**F**). (**A**,**B**)—Macroscopic presentation of oral melanoma: The tumor was located on the right side of the superior lip. It was multilobulated, poorly pigmented, and multifocally ulcerated; (**C**,**D**)—cytologic samples (MGG Quik Stain^®^ (04-090805; Bio Optica, Milano, Italy), 40× and 20× for (**C**) and (**D**) pictures, respectively) from the primary tumor and the lymph node, respectively. They show a population of neoplastic cells with marked anisocytosis and anisokaryosis. Sometimes, inside their cytoplasm, there is a finely granular greenish material (melanin); (**E**,**F**)—histology from the primary tumor and lymph node, respectively (hematoxylin–eosin, 40× and 10× for (**E**) and (**F**) pictures, respectively). The primary tumor appears densely cellular. The cells are arranged in lobules, supported by a moderate fibrous stroma. Neoplastic cells show a moderate amount of cytoplasm with poorly defined cell borders, round nuclei with vesicular chromatin, and prominent nucleoli.

**Figure 2:**
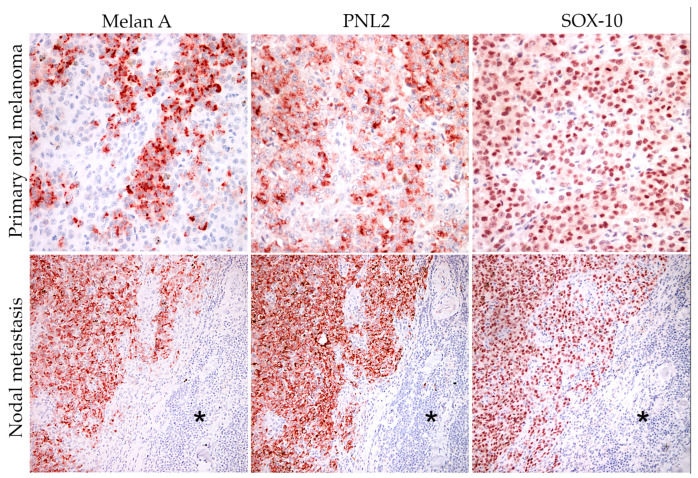
Case 3—Immunohistochemical expression of melanocytic markers (Melan-A, PNL2, and Sox-10) in both primary tumor (20×) and nodal metastasis (10×) (the asterisks represent the normal nodal tissue). The immunolabeling of Melan A and PNL2 was granular and cytoplasmic. Sox-10 was expressed in the nucleus of neoplastic cells. Carazzi’s hematoxylin was used as a counterstain.

Six cases were collected (anamnestic data and diagnosis are reported in Table 1). Three of the six cases were purebred (Pug, Shih-Tsu, and Miniature Pinscher), while the others were mixed breed (Figure 1); four dogs were males and two females. All dogs had a confirmed cytologic or histologic diagnosis of OMM with or without lymph nodal metastasis (Figure 1 and Table 1). For cases where the histopathology for diagnostic procedures was performed in our laboratories and, therefore, FFPE blocks were available (cases 2, 3, and 6), immunohistochemistry was performed with antibodies against Melan-A, PNL2, and Sox-10 [32,33,34] (Figure 2). Regarding sample collection for cell cultures, in three cases (cases 3, 5, and 6), FNA was performed on both the primary tumor and nodal metastasis. In one case (case 4), FNA was obtained only from the primary tumor, as the dog had no signs of nodal metastasis. Lastly, in two cases (cases 1 and 2), FNA samples were collected only from lymph node metastases because the primary tumor was not available due to prior surgical intervention by another practitioner.

### 3.2. Primary Cell Culture

**Figure 3:**
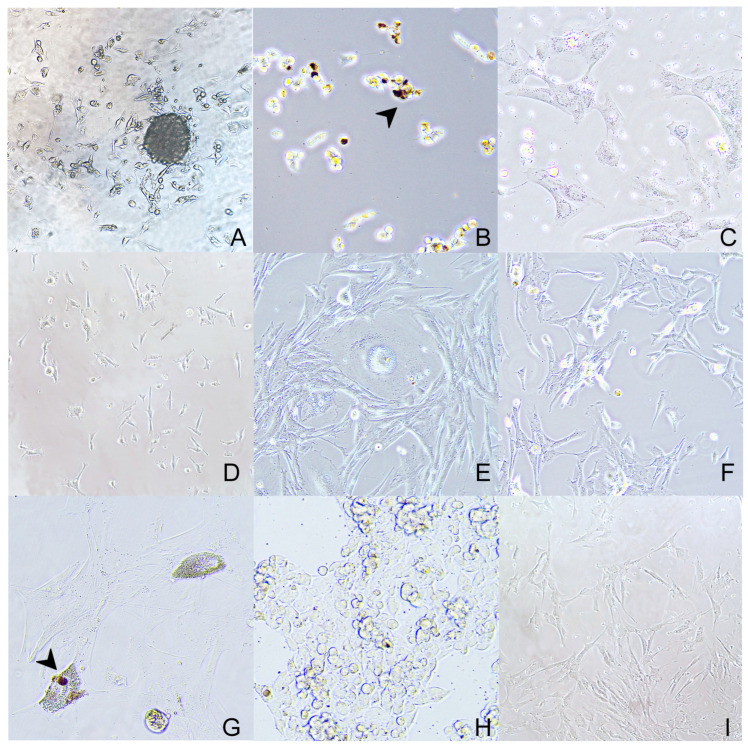
Two-dimensional cell cultures of neoplastic melanocytes obtained from primary oral neoplasia ((**A**) 20×—case 3; (**B**) 20×—case 4; (**C**) 40×—case 5; (**D**) 20×—case 6) and nodal metastasis ((**E**) 40×—case 1; (**F**) 40×—case 2; (**G**) 40×—case 3; (**H**) 40×—case 5, (**I**) 20×—case 6). The cells are often arranged in bundles, they are spindle-shaped with moderate cytoplasm, and well-defined cell borders that show dendritic prolongments. Within the cytoplasm of scattered cells, a dark brown finely granular pigment (melanin) is seen (arrowhead).

Two-dimensional primary cell cultures were obtained from all patients. Neoplastic cells were adherent after 24–48 h. Initially, neoplastic cells in culture were more polyhedral in shape; however, after the first passage, they achieved a more spindle/dendritic-shaped morphology. Highly pigmented tumor cell cultures showed a finely dark brown granulation (melanin pigment) in the cytoplasm (Figure 3). This pigment could also be observed free in the medium. Neoplastic cells occasionally showed cytoplasmatic optically empty vacuoles of variable size. Two-dimensional cell cultures of primary tumors were cultivated for 1 month, with a change of culture medium every 48 h, and remained vital for the whole period. Two-dimensional cell cultures of nodal metastases (1, 2, 3, and 6) yielded a confluency between 5 and 10 days and were stocked for further experiments.

**Figure 4:**
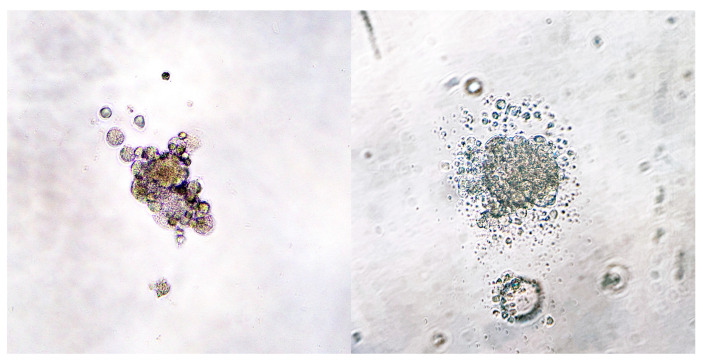
Case 1—Spheroids day 3 (40×). A thousand cells were seeded in a 96-well plate coated with 1.5% agar.

**Figure 5:**
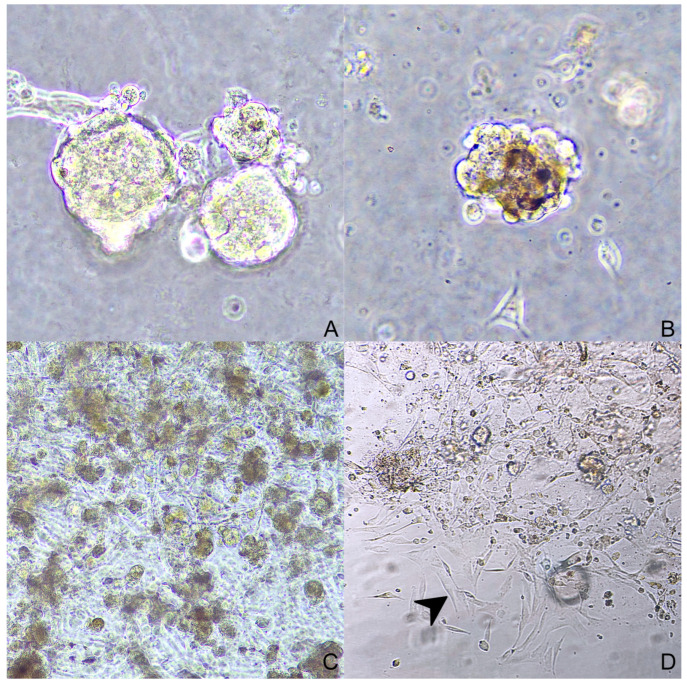
Organoids: ((**A**)—40×) case 1; ((**B**)—40×) case 4; ((**C**)—10×) case 2; ((**D**)—20×) case 3; in picture (**D**) neoplastic melanocytes are growing outside the Matrigel^®^, creating a two-dimensional culture (arrowhead).

Three-dimensional cell cultures were obtained in two ways. Spheroids were seeded from case 1 (nodal metastases) forming aggregates of round to polygonal neoplastic cells (Figure 4). Organoids were obtained and subsequently stored from cases 1, 2, 3, and 4 (nodal metastases in cases 1 and 2; primary tumors in cases 3 and 4). They were organized in multiple aggregates of different dimensions embedded in Matrigel^®^ (Figure 5). In some cases, a two-dimensional cell layer started to grow from the scaffold, occasionally reaching confluency (Figure 5D).

### 3.3. Cell Culture Characterization

**Figure 6:**
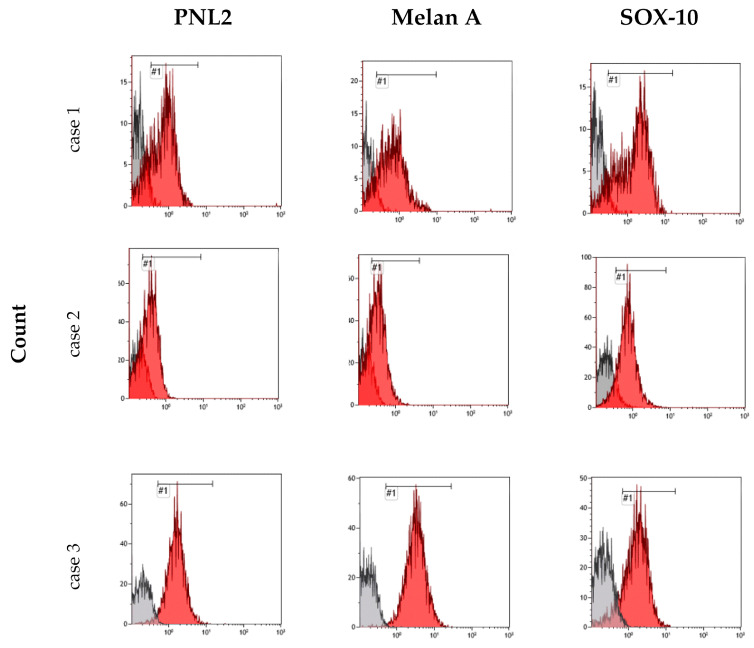
Flow cytometric analysis of cases 1, 2, and 3. The histograms show the different fluorescence intensity patterns for the tested markers: PNL2, Melan-A, and Sox-10. The red peaks represent the positive cells, while the grey peaks represent the negative ones. The sign #1 indicates the name of the gate.

**Figure 7:**
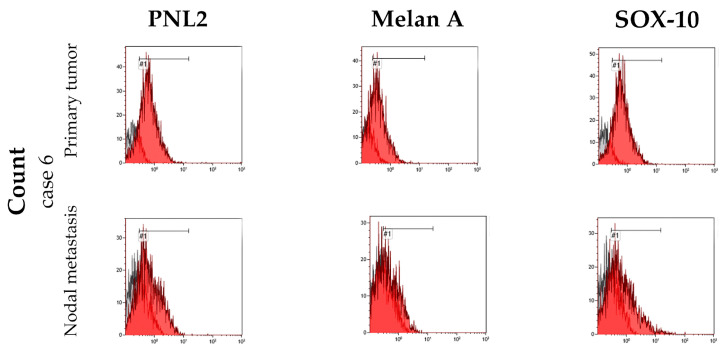
The histograms show the fluorescence intensity patterns for neoplastic melanocytes of primary tumor and lymph node metastasis marked with PNL2, Melan-A, and Sox-10 of case number 6. The red peaks represent the positive cells, while the grey peaks represent the negative ones. The sign #1 indicates the name of the gate.

During the flow cytometry analysis, cases 1, 2, 3, and 6 exhibited varying degrees of sensitivity to the permeabilizing treatment: case 1 showed signs of cellular stress visible from the morphological dot plot, while cases 2, 3, and 6 demonstrated satisfactory and normal morphological characteristics. The fluorescence peaks were well-defined, indicating the success of the labeling protocol, with a clear separation between the negative and the positive peaks. The fluorescence peaks, considering the area of the histogram representing positive fluorescence, were used to calculate the percentage of antibody-positive cells corresponding to the fluorescence (Figure 6 and Figure 7).

Case 1 presented consistent percentage values for the fluorescence of all three antibodies used, suggesting that over 70% of the isolated cells showed concurrent expression of Melan-A, PNL2, and Sox-10. Conversely, in case 2, a very high peak was observed for Sox-10, representing approximately 90% of the cells in the sample, contrasting with a discernible fluorescence representing only about 40% of cells for the Melan-A antigen and PNL2. Case 3 had clear and well-marked peaks representing 98% of the cells in the sample for Melan-A and PNL2, while positivity for Sox-10 was detected in only 79% of the cells. Case 6, on the other hand, exhibited a markedly distinct response compared to the previously analyzed cases: despite sharing similar characteristics with the other samples, it yielded positive signals for only a small percentage of cells for the three antibodies tested (Table 2).

**Figure 8:**
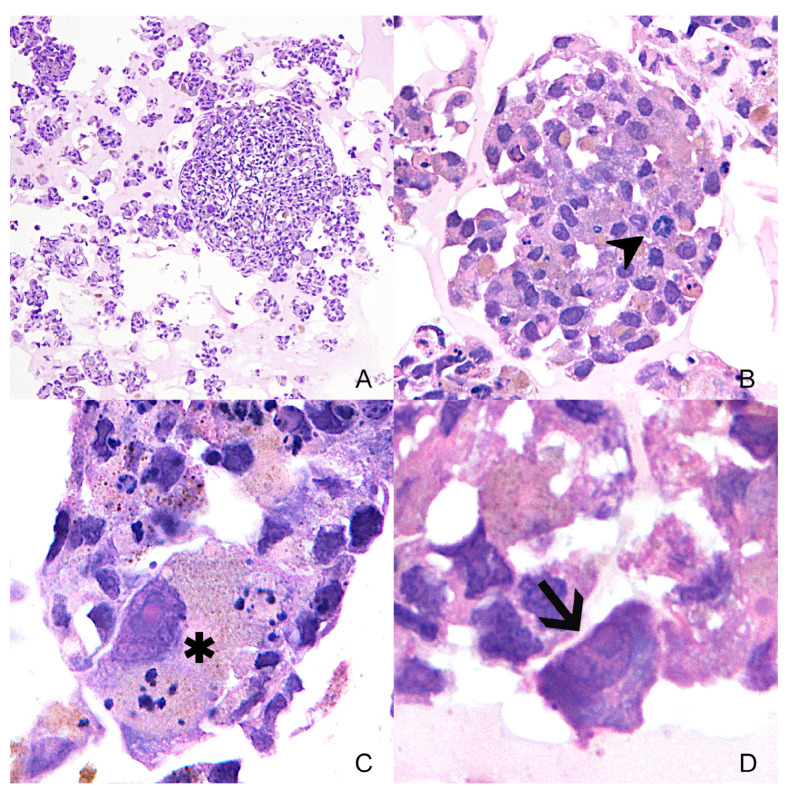
Variably size organoids ((**A**)—10×) of case 2 in hematoxylin and eosin. The cells show marked anisocytosis and anisokaryosis in all organoids. In Subfigure (**B**)—40×, mitotic figures are seen (arrowhead). In Subfigure (**C**)—100×, a cell with marked cytomegaly and karyomegaly is shown (asterisk). Subfigure (**D**)—100×, a high magnification of a cell showing multiple nucleoli (arrow).

Hematoxylin and eosin staining was performed on organoids of case 2 to evaluate their morphology. They showed variably sized aggregates of polygonal cells. The cells showed a moderate amount of cytoplasm, occasionally filled with finely granular, brown pigment. The nuclei were round to oval with single and prominent nucleoli. Cytomegaly was occasionally observed (Figure 8).

**Figure 9:**
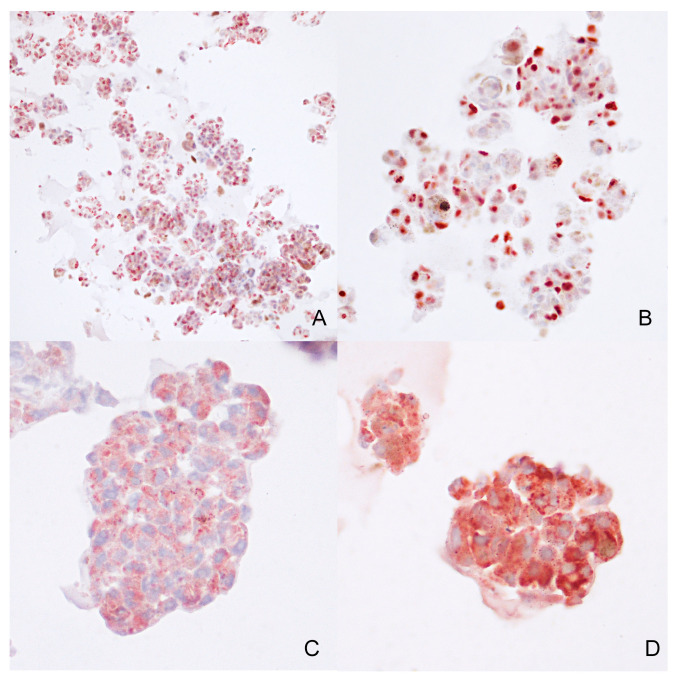
Case 2—Immunohistochemical expression of Sox-10 ((**A**)—10×), Ki-67 ((**B**)—20×), PNL2 ((**C**)—40×), TRP-1 ((**D**)—40×), in melanoma organoids.

Immunohistochemistry was conducted on the same FFPE tissue sample from case 2 to assess the phenotypical characteristics of the cells. Antibodies targeting PNL2, Sox-10, and TRP-1 exhibited widespread positivity (Figure 9), whereas Melan-A antibody positivity was limited. PNL2 and TRP-1 showed a diffuse cytoplasmatic positivity, while Sox-10 demonstrated a diffuse nuclear positivity. Additionally, Ki-67 nuclear immunolabeling was detected in approximately 80% of the cells.

## 4. Discussion

In this study, fine-needle aspiration was used as a method to obtain primary cell cultures from canine OMM and nodal metastases, as already reported in human medicine [28,40,41]. We successfully preserved a total of six distinct cell cultures, comprising two out of four derived from the primary oral tumor and four out of five obtained from lymph nodal metastasis. To the authors’ knowledge, this is the first study that reports primary tumor cell culture initiation from fine-needle aspirates in dogs.

Our study aimed to address a technological gap by providing a model for functional studies through the establishment of a method to obtain in vitro cell cultures using a non-invasive sampling technique. The cell lines obtained underwent comprehensive morphological and phenotypical characterization employing immunocytochemistry and flow cytometry, using antibodies against Melan-A, PNL2, and Sox-10. Future studies involving functional assays to assess the proliferative index of neoplastic cells and evaluate their sensitivity to specific agents in order to demonstrate the correlation between the behavior of primary tumor-derived cell cultures and the original tumors are planned by our research group.

Primary melanoma cell lines in dogs have been reported in veterinary medicine [42,43,44], yet their utilization and establishment are still relatively limited, particularly concerning organoids. As per the authors’ knowledge, organoids derived from canine OMM have not previously been established, highlighting the novelty of this study in the field of veterinary oncology.

Primary cultures are important to investigate molecular pathways and genetic mutations specifically involved in neoplastic cell transformation and phenotype definition. These alterations, often lost in immortalized cell lines due to multiple cell passages, are more robustly assessable in primary cultures. Therefore, our study primarily aimed at standardizing an easily accessible and relatively less invasive method for collecting tumor cells from dogs to establish primary cell cultures. FNA technique sampling is a widely used diagnostic tool, easily executable by veterinary practitioners, and minimally invasive for the patient. This method simplifies sample collection for in vitro model establishment from canine tumors, eliminating the need for immediate tissue processing in a sterile environment.

In particular, canine OMM can be challenging to sample for different reasons; first, oral melanomas are frequently non-eligible for surgery due to local extent or widespread dissemination. Moreover, owners may decline a demolitive oncological surgery due to associated prohibitive costs, poor prognosis, and potential compromise of anatomical functionality, alongside aesthetic concerns [8,45]. As a consequence, the proposed possibility of obtaining an FNA sample during routine diagnostic procedures could facilitate the collection of a larger number of samples without imposing additional risks or procedures on the patient.

Additionally, sampling primary oral tumors through the FNA technique could offer advantages over whole tissue sampling, particularly in these cases where tissue microbiota is abundant (i.e., canine oral mucosa or the gastrointestinal tract). As a matter of fact, canine OMMs are frequently ulcerated and harbor substantial bacterial overgrowth on the surface [46]. In our group’s experience, despite extensive curettage of excisional samples and repeated washing with antibiotics, cultures often remain susceptible to contamination. Conversely, direct aspiration with FNA from the inner portion of the tumor seems to mitigate this problem, providing cleaner samples.

However, despite its advantages, FNA has inherent limitations. Achieving an adequate cell yield for culturing often necessitated multiple tumor samplings from different parts of the lesion. Despite this, an adequate number of cells may not be reached consistently. This complicates the attainment of confluence and necessitates more passages to generate an adequate number of cells for experiments. Increased passages heighten the risk of molecular expression changes and accelerate aging of neoplastic cells. Moreover, blood contamination, particularly in samples collected from primary tumors, poses a technical challenge.

Our observations indicated a higher success rate in culturing cells obtained from lymph nodes compared to primary oral melanomas, likely due to the higher cellular concentration in the former. This was particularly evident in cases 1, 2, and 3, where samples from lymph node metastases yielded between 5 and 7 × 10^6^ cells/mL, reaching confluency more readily and in less time compared to primary oral melanoma. Moreover, metastatic tumor cells within lymph nodes might have acquired motility by losing intercellular junctions [47,48], therefore accounting for an easier exfoliation, limiting cell stress and damage

In this study, we sought to explore the feasibility of establishing three-dimensional (3D) cell cultures from FNA-derived samples of canine OMMs. Working with 3D cell cultures better mirrors cell–cell and cell–microenvironment interactions, providing a closer representation of tumor heterogeneity and the microenvironment [49].

Nowadays, cancer therapy and immunotherapy are being increasingly used in veterinary medicine [7,14]; therefore, despite the aforementioned challenges, the proposed method exploiting FNA may represent an innovative tool to expedite and broaden oncology studies in dogs and pets in general, providing accessible 3D models. These models, particularly organoids, represent a more faithful in vitro representation compared to 2D cell cultures and could serve as an essential platform to investigate the interaction between neoplastic and co-cultured immune cells in a 3D setting, providing additional insights into the immune environment. In addition, a broader array of in vitro models might provide more robust comparative data for the rare human mucosa melanoma. Finally, in a perspective application of personalized medicine in veterinary medicine, these methods might be a useful asset.

## 5. Conclusions

To the best of the authors’ knowledge, this research represents the first utilization of fine-needle aspiration to establish both two-dimensional and three-dimensional primary neoplastic cell cultures from dogs. This study provides a reliable method for initiating primary cell culture, significantly expanding the study of canine melanocytic tumors as a model for their human counterparts. In an era where personalized medicine is becoming increasingly required, the use of primary cell cultures derived from fine-needle aspiration offers a faster and more effective means to test and provide substantial support in cancer therapy.

## Figures and Tables

**Table 1:** Anamnestic data, sampling site, and cytologic or histologic diagnosis. Age is reported in years; F indicates female, while M indicates male. Diagnoses marked with an asterisk (*) indicate cases diagnosed in our laboratory. Absence of an asterisk indicates diagnoses performed in another laboratory. The sign ✓ indicates the presence of the sample.

Case	Dog			Tumor				
	Breed	Age	Sex	Sampling Site		Diagnosis		
				Primary Tumor	Lymph Node	Cytology	Histology	Immunohistochemistry
1	Pug	14	F		✓	✓		
2	Miniature Pinscher	12	F		✓	✓*	✓*	✓
3	Mixed breed	12	M	✓	✓	✓*	✓*	✓
4	Shih-Tsu	12	M	✓		✓	✓	
5	Mixed breed	10	M	✓	✓	✓		
6	Mixed breed	12	M	✓	✓	✓*	✓*	✓

**Table 2:** Percentage of positive cells in flow cytometric analysis of cases 1, 2, 3, and 6 (^a^ = primary tumor; ^b^ = nodal metastasis).

Antigen	Percentage of Positive Cells				
	Case 1	Case 2	Case 3	Case 6 ^a^	Case 6 ^b^
PNL2	66.6	53.4	95.8	74	43.8
Melan-A	73.9	53.4	99	48.2	19.2
Sox-10	85	82	78.5	79.2	39.6

## Data Availability

The data presented in this study are available upon request from the corresponding author.
